# Muscarinic Receptor Antagonism and TRPM3 Activation as Stimulators of Mitochondrial Function and Axonal Repair in Diabetic Sensorimotor Polyneuropathy

**DOI:** 10.3390/ijms26157393

**Published:** 2025-07-31

**Authors:** Sanjana Chauhan, Nigel A. Calcutt, Paul Fernyhough

**Affiliations:** 1 Division of Neurodegenerative & Neurodevelopmental Disorders, St. Boniface Hospital Albrechtsen Research Centre, University of Manitoba, Winnipeg, MB R2H 2A6, Canada; chauhans@myumanitoba.ca; 2 Department of Pharmacology and Therapeutics, Rady Faculty of Health Sciences, Max Rady College of Medicine, University of Manitoba, Winnipeg, MB R3E 0T6, Canada; 3 Department of Pathology, University of California San Diego, La Jolla, CA 92093, USA; ncalcutt@health.ucsd.edu

**Keywords:** bioenergetics, Ca^2+^ homeostasis, diabetic neuropathy, DRG, GPCR, pirenzepine

## Abstract

Diabetic sensorimotor polyneuropathy (DSPN) is the most prevalent complication of diabetes, affecting nearly half of all persons with diabetes. It is characterized by nerve degeneration, progressive sensory loss and pain, with increased risk of ulceration and amputation. Despite its high prevalence, disease-modifying treatments for DSPN do not exist. Mitochondrial dysfunction and Ca^2+^ dyshomeostasis are key contributors to the pathophysiology of DSPN, disrupting neuronal energy homeostasis and initiating axonal degeneration. Recent findings have demonstrated that antagonism of the muscarinic acetylcholine type 1 receptor (M_1_R) promotes restoration of mitochondrial function and axon repair in various neuropathies, including DSPN, chemotherapy-induced peripheral neuropathy (CIPN) and HIV-associated neuropathy. Pirenzepine, a selective M_1_R antagonist with a well-established safety profile, is currently under clinical investigation for its potential to reverse neuropathy. The transient receptor potential melastatin-3 (TRPM3) channel, a Ca^2+^-permeable ion channel, has recently emerged as a downstream effector of G protein-coupled receptor (GPCR) pathways, including M_1_R. TRPM3 activation enhanced mitochondrial Ca^2+^ uptake and bioenergetics, promoting axonal sprouting. This review highlights mitochondrial and Ca^2+^ signaling imbalances in DSPN and presents M_1_R antagonism and TRPM3 activation as promising neuro-regenerative strategies that shift treatment from symptom control to nerve restoration in diabetic and other peripheral neuropathies.

## 1. Introduction

Diabetes mellitus (DM) is a significant metabolic disorder with profound global health implications. Impaired insulin signaling, arising from either loss of insulin (type 1 diabetes) or insulin resistance (type 2 diabetes), is central to the pathophysiology of DM [1,2]. Chronic hyperglycemia, a hallmark of both forms of DM, contributes to long-term damage, dysfunction, and failure of various organs including the retina, kidneys, peripheral nerves, cardiovascular system, and blood vessels [1,2]. Dyslipidemia is increasingly recognized as another primary pathogenic insult, particularly in the context of obesity, prediabetes and type 2 diabetes [3]. In this review, we describe and assess the therapeutic potential of muscarinic acetylcholine type 1 receptor (M_1_R) antagonism and transient receptor potential cation channel subfamily M member 3 (TRPM3) activation, two interconnected pathways that control Ca^2+^ regulation, neuronal metabolism and axonal outgrowth. By integrating basic research findings with current clinical efforts, including ongoing clinical trials, this review highlights new directions for disease-modifying treatments of DSPN and related peripheral neuropathies that go beyond symptomatic relief to augment mitochondrial function and promote nerve repair.

## 2. Diabetic Sensorimotor Polyneuropathy

DSPN is a significant complication of DM, affecting up to 50% of individuals with the disease and profoundly impacting their quality of life [1,4]. Primary consequences of diabetes that drive DSPN include loss of insulin signaling arising from insulinopenia or insulin resistance and chronic hyperglycemia. The resulting cascade of biochemical disturbances promotes diverse neurotoxic insults such as deficits in neurotrophic support and the accumulation of advanced glycation end products (AGEs) and reactive oxygen species (ROS) [1,5,6]. These processes collectively disrupt neuronal integrity and function, ultimately manifesting as sensory loss, motor dysfunction and, in some cases, pain [7]. DSPN typically presents with a “stocking-glove” distribution of sensory deficits, with the hands and feet initially affected then progressing proximally over time through a dying-back process of axonal degeneration [1,2]. This loss of sensation compromises balance and the ability to detect injuries, increasing the risk of foot ulcers which, if not properly managed, can lead to infections and ultimately limb amputations [8].

Despite the widespread prevalence of DSPN, this neuropathy is often underdiagnosed, largely due to variable endpoint measurement methods, differing DSPN definitions, and the diverse types of patients studied [9]. Neurological signs and electrophysiological measurements are widely used as analytical tools in diagnosing DSPN [10], yet the lack of standardized criteria complicates the identification of the condition. The risk and severity of DSPN are closely correlated with the duration of diabetes and the degree of glycemic control, highlighting the importance of stringent blood glucose management to mitigate progression [11]. Consequently, current treatment strategies focus primarily on glycemic control and, where applicable, adjuvant pain management. However, these approaches are not always effective in halting the progression of DSPN, particularly in patients with type 2 diabetes [12], underscoring the urgent need for early diagnosis and therapeutic options. Currently, there is no disease-modifying therapy available to patients with DSPN. Furthermore, DSPN often coexists with autonomic neuropathy, another underdiagnosed condition, leading to abnormalities in cardiovascular, gastrointestinal, and genitourinary function [13]. This autonomic involvement augments the complexity of neuropathic damage, via impacts on blood flow and homeostatic balance, thereby aggravating nerve degeneration.

## 3. Epidemiology—Economic and Social Burden of DSPN

DSPN represents a serious and expanding global health challenge that is intricately linked to the escalating prevalence of diabetes mellitus [9,14]. The International Diabetes Federation (IDF) reports that, as of 2021, approximately 537 million adults aged 20–79 years were living with diabetes, equating to 1 in 10 adults globally [15]. This number is expected to rise to 643 million by 2030 and 783 million by 2045. Over three-quarters of these individuals reside in low and middle-income countries where healthcare infrastructure may be less capable of effectively managing chronic complications such as DSPN. The annual global expenditure on treating diabetic neuropathy and its complications is correspondingly enormous, with up to 27% of the direct medical cost of diabetes attributed to DSPN [16]. For example, a recent analysis estimated that the annual cost burden of the 13 million persons with diabetic neuropathy in the USA was almost US $46 billion, including over $30 billion for direct inpatient and outpatient care [17].

## 4. Modeling DSPN

In DSPN the dorsal root ganglia (DRG) and associated peripheral nerves undergo significant pathological changes [5]. Animal models replicate several features of early nerve pathology observed in humans, including reduced density of epidermal and corneal small sensory nerve fibers, myelin thinning, and a reduction in the size of large myelinated axons [18,19,20,21,22]. However, despite these parallels, the most commonly used rodent models often fall short in replicating the late-stage structural pathology of human DSPN, particularly the extensive loss of fibers, Schwann cell pathology and segmental demyelination [20,23]. The variability in neuropathy reported amongst different models highlights the intricate and varied nature of DSPN pathogenesis and progression, which can be influenced by factors such as background strain, diet composition, insulin/C-peptide deficiency, and coexisting conditions such as dyslipidemia and hypertension [3,24,25]. Despite these limitations, rodent models provide a controlled environment to explore early molecular events that cannot be studied in humans. Additionally, advancements in nerve imaging techniques, such as corneal confocal microscopy (CCM) and intraepidermal nerve fiber (IENF) density measurements, have enhanced our ability to monitor small fiber neuropathy in these models and to compare directly with the human condition [26,27].

## 5. Pathogenesis of DSPN: Crosstalk Between Metabolic Pathways

**Figure 1:**
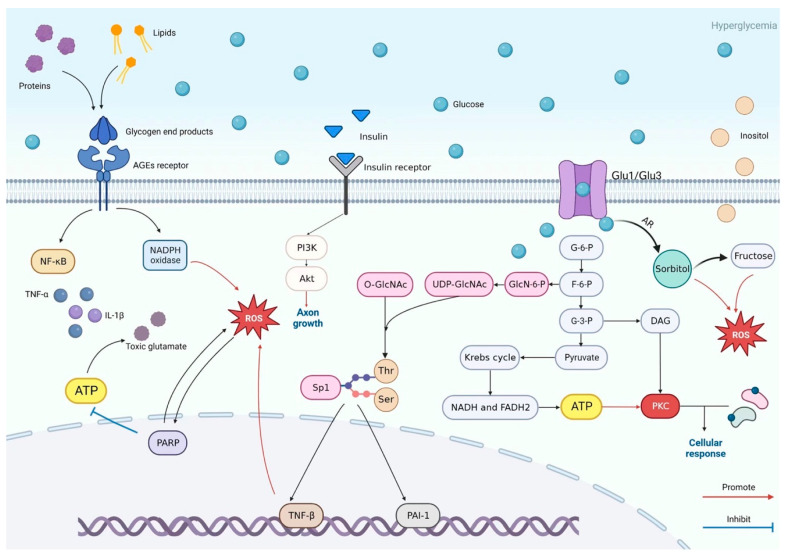
Hyperglycemia-driven pathways contributing to DSPN. Under hyperglycemic conditions, excess glucose passively enters cells through glucose transporters and is shunted into multiple metabolic pathways. Glucose is converted into sorbitol and fructose via the polyol pathway, generating reactive oxygen species (ROS) and redox imbalance. Increased glucose also promotes the formation of advanced glycation end-products (AGEs), activating their receptor and stimulating NADPH oxidase, NF-κB, and pro-inflammatory cytokines. The hexosamine pathway modifies transcription factors (e.g., Sp1), altering gene expression of factors such as TNF-β. Elevated diacylglycerol (DAG) activates protein kinase C (PKC), influencing cellular responses. Collectively, these intertwined mechanisms lead to oxidative stress, inflammation, and disrupted cellular function. Reprinted/Adapted from ref. [34].

Peripheral neurons that innervate the feet, are the longest cells in the human body. These sensory and motor neurons are heavily reliant on a robust vascular supply, functional mitochondria, and tightly regulated glucose and lipid metabolism to meet their substantial energy requirements [28,29]. Neurons, even in a quiescent state, demand a constant energy supply to sustain membrane potential, propagate action potentials, and recycle neurotransmitters [30]. Neurons primarily uptake glucose via the insulin-independent glucose transporter 3 (GLUT3) [31], after which it is metabolized via glycolysis into pyruvate. However, in diabetes, these tightly regulated metabolic pathways can become overwhelmed by excess glucose (Figure 1), leading to the activation of auxiliary metabolic routes, such as the polyol and hexosamine pathways [32]. Hyperglycemia also results in the non-enzymatic binding of glucose to amino residues of various structural and functional proteins, leading to the formation of advanced glycation end-products (AGEs), excess of which are detected in the nerves of diabetic patients and animal models of diabetes [33].

In type 2 diabetes, both hyperlipidemia and dyslipidemia exacerbate DSPN in conjunction with hyperglycemia. Under normal physiological conditions, long-chain fatty acids enter the β-oxidation pathway, with each cycle producing one molecule of acetyl-CoA. However, in diabetes, excessive substrate supply overwhelms mitochondrial capacity, resulting in incomplete fatty acid oxidation and the accumulation of intermediary byproducts, such as acylcarnitines [35]. These toxic metabolites contribute to mitochondrial dysfunction, impairing ATP production and increasing reactive oxygen species (ROS) levels, which in turn damage neurons and Schwann cells [3,36,37]. In addition, serine metabolism is suppressed and can drive nerve damage [38]. Dyslipidemia, marked by elevated triglyceride and LDL cholesterol levels and reduced HDL cholesterol, further promotes serine depletion, vascular inflammation and oxidative stress, impairing blood flow and activating pro-inflammatory pathways in the peripheral nervous system [3,38]. The complex role of inflammatory cells, acting as both protective and damaging influences, has been recently highlighted. Thus, in a prediabetic mouse model, the inhibition of macrophage recruitment and subsequent activation in the sciatic nerve via genetic deletion of macrophage genes *CCR2* or *Lgals2*, enhanced heat hypoalgesia and IENF loss [39]. Further, hyperglycemia and subsequent enhanced glucose uptake by mast cells triggered their degranulation and release of inflammatory mediators that contributed to the development of neuropathy while development of diabetic neuropathy was suppressed by mast cell deficiency [40].

## 6. Molecular and Cellular Mechanisms Driving DSPN

### 6.1. Mitochondrial Dysfunction in DSPN Pathology

**Figure 2:**
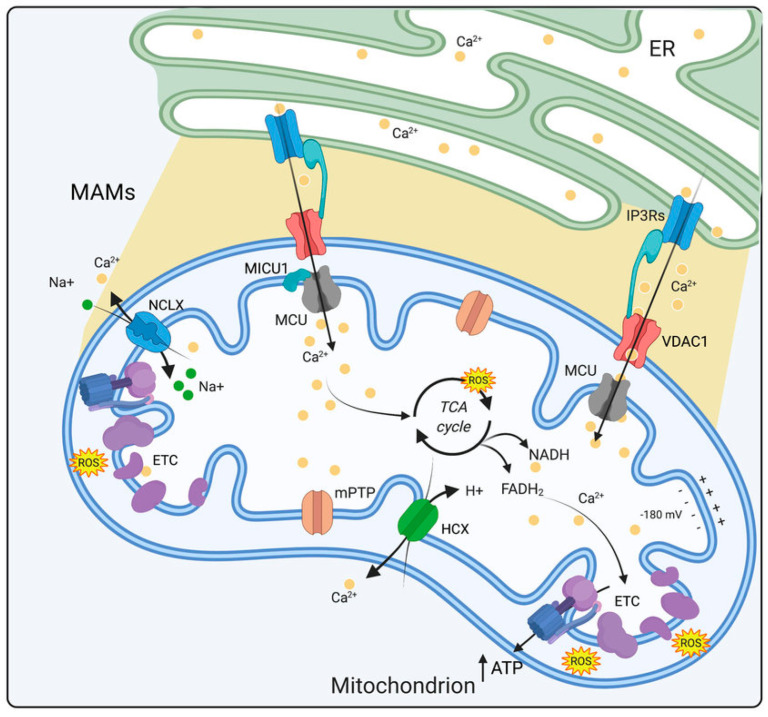
Mitochondrial Ca^2+^ homeostasis. Mitochondrial Ca^2+^ homeostasis is tightly regulated by influx and efflux mechanisms. Ca^2+^ enters the mitochondrial matrix via the MCU and through a high electronegative potential (−180 mV) while its extrusion depends on NCLX and HCX exchangers. Within the matrix, Ca^2+^ stimulates the activity of three dehydrogenases of the Krebs cycle and ATP production. Ca^2+^ ions are depicted as yellow dots. Abbreviations: ER, endoplasmic reticulum; MAMs, mitochondria associated membranes; ETC, electron transport chain; MCU, mitochondrial Ca^2+^ uniporter; VDAC1, voltage-dependent anion channel 1; ATP, adenosine triphosphate; MICU1, mitochondrial Ca^2+^ uptake 1; IP3Rs, inositol-1,4,5-trisphosphate receptors; ROS, reactive oxygen species; mPTP, mitochondrial permeability transition pore; NCLX, Na^+^/Ca^2+^ exchanger; HCX, H^+^/Ca^2+^ exchanger. Reprinted/Adapted from ref. [46].

Mitochondrial dysfunction is a well-established feature of DSPN, with structural abnormalities consistently documented across multiple animal models and human studies [41,42]. Mitochondria, as dynamic organelles, are vital for several cellular processes, most notably ATP generation through oxidative phosphorylation, but also via roles in Ca^2+^ homeostasis and regulation of apoptotic pathways [43]. Their structural integrity, defined by a double membrane system comprising the outer and inner membranes, maintains cellular energy homeostasis. The outer membrane contains voltage-dependent anion channels (VDACs), which allow for the exchange of ions and small molecules, while also playing roles in lipid synthesis and the import of mitochondrial precursors [44] (Figure 2). In contrast, the inner membrane contains transport proteins that regulate metabolite exchange and also houses the electron transport chain (ETC), which is integral to ATP production [45].

In DSPN, mitochondria exhibit a complex and heterogeneous response to stressors, varying by cell type and affected regions. In rodent models of type 1 diabetes, such as streptozotocin (STZ)-diabetic and BioBreeding (BB) rats, mitochondria of Schwann cells display significant ultrastructural abnormalities, while mitochondria of axons and neuronal perikarya appear relatively normal [47,48]. There is also an accumulation of small, hyperdense mitochondria within sympathetic neurons [49,50,51]. These smaller mitochondria often aggregate in post-synaptic dendrites, forming tightly packed clusters without intervening cytoplasm, indicative of increased mitochondrial fission in response to the diabetic environment [52]. In contrast, models of type 2 diabetes, such as the db/db mouse, display an increased density of mitochondria in sensory axons, yet these mitochondria appear to have a normal ultrastructure [48]. Mitochondrial proliferation may occur as a compensatory mechanism in response to metabolic stress, without overt structural damage [53].

At a functional level, respiratory chain activity of mitochondria is markedly reduced in DSPN [54,55,56]. Depolarization of the mitochondrial inner membrane has been observed in sensory neurons that were acutely isolated from DRG of adult STZ-diabetic rats [57,58]. Work by ourselves and the Dobrowsky laboratory has employed various methodologies to demonstrate that mitochondrial respiratory chain activity is diminished in sensory neurons derived from DRG of both type 1 and type 2 diabetic rodents [54,59,60]. Further investigation into mitochondrial physiology has revealed that ATP synthase inhibition via oligomycin administration results in significant hyperpolarization of the mitochondrial inner membrane in neurons from diabetic rats compared to controls [59]. In healthy neurons, ATP synthase inhibition typically induces a transient hyperpolarization of the mitochondrial inner membrane, followed by a compensatory response facilitated by uncoupling proteins (UCPs). However, neurons from diabetic animals exhibit a prolonged hyperpolarization of the mitochondrial inner membrane followed by an impaired recovery phase, suggesting an inability to regulate mitochondrial membrane potential effectively [61]. This maladaptive response may stem from insufficient expression or functional impairment of UCPs, leading to suboptimal ROS modulation and increased susceptibility to oxidative damage [62]. Studies using a mouse model of pre-diabetes induced by high-fat diet (HFD) have demonstrated that lipid-mediated mitochondrial dysfunction in the early stages of peripheral neuropathy plays a key role reducing membrane potential and bioenergetic reserve capacity [37]. This is accompanied by decreased intra-axonal Ca^2+^, mitochondrial elongation, and upregulation of peroxisome proliferator-activated receptor gamma coactivator-1α (PGC1α), suggesting a compensatory but inadequate response to lipid-induced mitochondrial stress.

Mitochondrial dysfunction has also been investigated in humans with DSPN. Immunostaining of skin biopsies revealed a significant reduction in the expression of Complex IV components in intraepidermal and subpapillary dermal nerve fibers when compared to tissue from non-diabetic subjects [63]. This reduction in Complex IV expression occurred before significant fiber loss, suggesting that mitochondrial dysfunction may precede overt neurodegeneration. However, contradictory findings emerged from a 3D imaging study, which demonstrated enhanced mitochondrial volumes within IENFs of patients with diabetic neuropathy [64]. This discrepancy may reflect different stages of DSPN progression, where early-stage disease is characterized by reduced mitochondrial function, followed by a compensatory increase in mitochondrial volume in response to elevated energy demands during later stages of disease.

### 6.2. Ca^2+^ Imbalance in Progression of DSPN

Ca^2+^ dyshomeostasis, in which the finely tuned regulation of intracellular Ca^2+^ is profoundly disrupted, is a hallmark pathophysiological mechanism associated with neuronal dysfunction in diabetes [65]. Ca^2+^ ions act as intracellular second messengers, regulating a range of neuronal processes including neurotransmission, excitability, and cell survival [66]. Under normal conditions, cellular Ca^2+^ homeostasis is tightly regulated by a coordinated system of Ca^2+^ channels, transporters, and buffers across various intracellular compartments, notably the cytosol, endoplasmic reticulum (ER), and mitochondria [67]. However, the diabetic state impairs this intricate regulatory network, leading to aberrant Ca^2+^ homeostasis and contributing to neuronal degeneration.

The ER plays a central role in maintaining Ca^2+^ balance [68]. It acts as the primary intracellular Ca^2+^ store, sequestering Ca^2+^ via the action of sarco/endoplasmic reticulum Ca^2+^ ATPase (SERCA) pumps. These pumps are responsible for transporting Ca^2+^ from the cytosol into the ER lumen, where the concentration of free Ca^2+^ reaches millimolar levels (0.5–1.0 mM). This Ca^2+^ reserve is released in response to various physiological stimuli, with inositol 1,4,5-trisphosphate receptors (IP_3_Rs) and ryanodine receptors (RyRs) mediating Ca^2+^ release to the cytosol, thereby activating a cascade of Ca^2+^-dependent processes vital for neuronal activity, such as neurotransmitter release and long-term potentiation.

In sensory neurons from diabetic animals, the ability of the ER to release Ca^2+^ via IP_3_Rs and RyRs is significantly compromised, leading to a reduction in Ca^2+^ transients essential for rapid neuronal responses [69]. This impairment in Ca^2+^ signaling is exacerbated by the decrease in SERCA activity, which slows the reuptake of Ca^2+^ into the ER following its release. As a result, there is a sustained elevation in cytosolic Ca^2+^ concentration ([Ca^2+^]i), which triggers maladaptive cellular responses. Prolonged elevations in [Ca^2+^]i lead to the activation of Ca^2+^-dependent enzymes, such as calpains and caspases, which degrade cytoskeletal proteins and initiate neurodegenerative signaling cascades [66]. Mitochondria are also key players in cellular Ca^2+^ regulation, being responsible for buffering cytosolic Ca^2+^ during periods of high neuronal activity via Ca^2+^ uptake through the mitochondrial Ca^2+^ uniporter (MCU) [70] (Figure 2). This Ca^2+^ influx into mitochondria regulates metabolic enzymes within the Krebs cycle, which in turn enhances ATP production to meet the heightened energy demands of neurons [71]. In DSPN, mitochondrial Ca^2+^ handling is impaired, leading to Ca^2+^ overload within the organelle [72].

Molecular chaperones provide an essential layer of defense against cellular functional collapse. Beyond their role in the ER, cytosolic heat shock proteins (HSPs) such as Hsp70 and Hsp90 have emerged as powerful modulators of cellular stress in the nervous system of diabetic rodents [56]. Modulating Hsp70 expression enhances neuronal resilience to glucotoxic insults and improves mitochondrial function [73]. Similarly, the inhibition of Hsp90 at its C-terminal domain by agents such as KU-32 has demonstrated the potential to restore mitochondrial bioenergetics, alleviate oxidative stress, and preserve sensory nerve function in rodent models of diabetes [74]. The dysfunction of mitochondria in DSPN is also closely linked to disruption of Ca^2+^ signaling at mitochondria-associated membranes (MAMs), the specialized contact sites between the ER and mitochondria [75]. MAMs facilitate the efficient transfer of Ca^2+^ from the ER to mitochondria (Figure 2). Diabetes impairs the function of MAMs, leading to impaired Ca^2+^ transfer and a further reduction in mitochondrial ATP production [76].

The disturbance of Ca^2+^ homeostasis within sensory neurons extends to the plasma membrane, where voltage-gated Ca^2+^ channels mediate Ca^2+^ entry in response to neuronal depolarization. Alterations in the expression and function of these channels have been observed in animal models of DSPN, with increased density of both low- and high-threshold Ca^2+^ currents reported in sensory neurons [77]. However, despite this increase in channel density, depolarization-induced Ca^2+^ transients are often depressed, particularly in neurons with long axons, such as those projecting from the lumbar DRG [78,79]. This paradoxical reduction in Ca^2+^ transients may be attributed to enhanced Ca^2+^-dependent inactivation of Ca^2+^ channels, a process likely exacerbated by elevated resting [Ca^2+^]i levels in neurons from diabetic animals.

## 7. AMP-Activated Protein Kinase (AMPK)

### 7.1. AMPK Structure and Function

**Figure 3:**
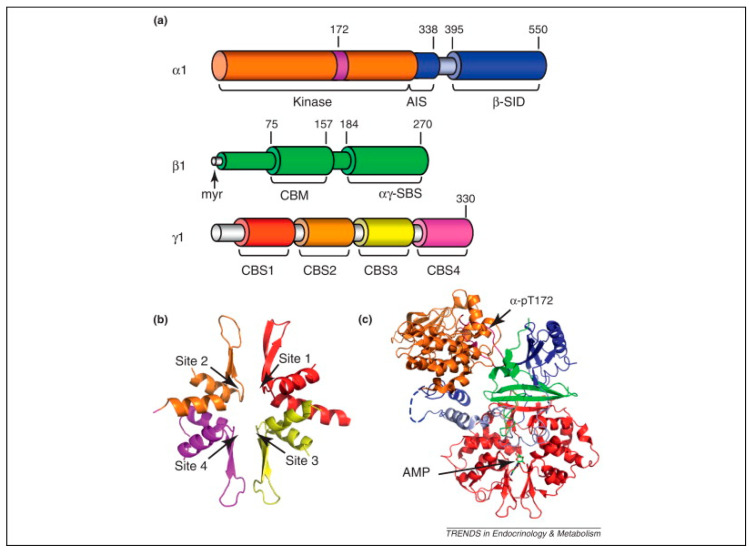
AMPK domains and structure. (**a**) Domain organization of AMPK subunits. Residue numbering refers to human α1, β1 and γ1 isoforms. The α subunit consists of an N-terminal kinase domain, an autoinhibitory sequence [83] and a β-subunit interacting domain (β-SID). The β subunit is N-terminally myristoylated (myr) and contains a mid-molecule carbohydrate-binding module (CBM) and C-terminal αγ subunit-binding sequence (SBS). The γ subunit contains four cystathione β-synthase (CBS) domains, paired (1+2 and 3+4) to form two Bateman modules. (**b**) Tetrad organization of CBS domains in the γ-subunit, colored as in (**a**), showing locations of nucleotide binding sites (black arrows). (**c**) Structure of the mammalian AMPK regulatory core and kinase domain [PDB 2Y94: rat α1 (7–299)/(331–469)/(524–548), human β1 (198–272) (green), rat γ1 (23–326) (red)]; α-subunit regions are colored as in (**a**). AMP bound at γ site 3 is evident. Reprinted/Adapted with permission from ref. [84]. 2012 Jonathan S. et al.

The Ca^2+^ sensitive AMP-activated protein kinase (AMPK) functions as a central metabolic sensor, orchestrating cellular responses to energy stress by modulating a variety of biochemical processes. Structurally, AMPK is a heterotrimer composed of a catalytic α subunit (α1 or α2) and regulatory β (β1 or β2) and γ subunits (γ1, γ2, or γ3), with the γ subunit containing four cystathione β-synthase (CBS) repeats that bind AMP, ADP, and ATP [80] (Figure 3). This structural arrangement allows AMPK to detect shifts in cellular energy status, particularly changes in the ATP/AMP ratio, and subsequently adjust its activity [81]. Under conditions of diminished ATP, AMP binds competitively to the CBS domains, enhancing basal AMPK activity and facilitating phosphorylation of the α subunit at Thr172 by upstream kinases, such as liver kinase B1 (LKB1) [82]. Phosphorylation at this site increases AMPK activity by approximately 100-fold, triggering a cascade of downstream effects that collectively promote catabolic pathways to restore ATP levels while concurrently suppressing anabolic processes that consume energy [82]. Once activated, AMPK initiates metabolic shifts by phosphorylating key enzymes across multiple pathways that maneuver cellular metabolism towards an energy-conserving mode supporting ATP regeneration.

AMPK plays a key role in mitochondrial biogenesis, primarily through its interaction with PGC-1α, a transcriptional coactivator that stimulates the transcription of mitochondrial genes [85]. PGC-1α enhances mitochondrial DNA replication and activates nuclear respiratory factors (NRF1 and NRF2), which in turn stimulate mitochondrial transcription factor A (mtTFA) to drive mitochondrial DNA transcription [86]. Phosphorylation of PGC-1α by AMPK enhances this pathway, leading to increased mitochondrial biogenesis and oxidative phosphorylation capacity [55,87,88].

### 7.2. AMPK Abnormalities in DSPN

In DSPN, AMPK signaling within the DRG is downregulated and this has been linked to mitochondrial dysfunction and subsequent neuropathy [55]. In DRG of diabetic rodents, reduced AMPK phosphorylation correlates with mitochondrial depolarization, diminished mitochondrial biogenesis, and increased vulnerability to neuronal injury [55,61,89]. Therapeutic strategies to restore AMPK activity, such as the administration of resveratrol, reverse these defects by enhancing AMPK signaling, thereby promoting mitochondrial polarization and stimulating axonal outgrowth [55,90]. Resveratrol has also demonstrated efficacy in mitigating oxidative stress, which is a central pathological feature in diabetic neuropathy [91]. In STZ-diabetic rats, resveratrol treatment significantly ameliorated indices of neuropathy including reduced nerve conduction velocity, reduced nerve blood flow and hyperalgesia to heat. These neuroprotective effects were associated with decreased levels of oxidative stress markers, such as malondialdehyde (MDA) and peroxynitrite, alongside increased catalase activity. Resveratrol also targets inflammatory pathways by inhibiting the nuclear factor kappa-light-chain-enhancer of activated B cells (NF-κB) signaling cascade, which is upregulated in diabetic neuropathy in association with oxidative stress and AGE formation [92]. NF-kB inhibition reduces pro-inflammatory mediators such as cyclooxygenase-2 (COX-2), TNF-α, and interleukin-6 (IL-6), while also decreasing oxidative damage markers such as MDA, further highlighting the neuroprotective potential of AMPK enhancers against diabetic neuropathy. Pharmacological activators of AMPK, such as metformin and AICAR, have also been reported to improve nerve conduction velocity, reduce inflammation and normalize mitochondrial function in animal models of diabetes, further supporting a potential therapeutic application for AMPK activators in DSPN [93,94].

AMPK is also activated by Ca^2+^/calmodulin-dependent protein kinase kinase β (CaMKKβ) [95,96], which is of particular interest due to its Ca^2+^ dependency. Unlike LKB1, which is primarily activated by changes in the AMP ratio, CaMKKβ responds to increases in intracellular Ca^2+^ levels, allowing AMPK to be activated independently of cellular energy status [97]. This mechanism is especially pertinent in neurons, where Ca^2+^ fluctuations are frequent and indispensable for various signaling cascades. CaMKKβ-mediated AMPK activation enables cells to respond rapidly to changes in intracellular Ca^2+^, thereby supporting essential functions such as mitochondrial biogenesis and the maintenance of mitochondrial function. In this capacity, AMPK promotes axonal outgrowth, providing a neuroprotective effect in environments where energy homeostasis is compromised, such as in diabetes [55,98].

## 8. Role of M_1_R in Nerve Repair

### 8.1. GPCR Pharmacology

G protein-coupled receptors (GPCRs) form one of the most extensive and varied families of membrane proteins, acting as vital mediators of cellular communication [99]. GPCRs are activated by a broad range of ligands, including neurotransmitters, hormones, and other signaling molecules, making them indispensable for maintaining cellular homeostasis [100,101]. Among GPCRs, the muscarinic acetylcholine receptors (mAChRs) stand out as a highly conserved class of GPCR that function in the central and peripheral nervous systems [102]. These receptors are divided into five subtypes: M1, M2, M3, M4, and M5. Each subtype is distinguished by its unique G protein-coupling preferences. The M1, M3, and M5 subtypes are primarily associated with Gq/11 proteins, leading to the activation of phospholipase C (PLCβ), which subsequently increases intracellular Ca^2+^ signaling. In contrast, M2 and M4 receptors couple with Gi/o proteins, which inhibit adenylyl cyclase and reduce cyclic AMP levels, creating distinct physiological effects across various tissues [103]. Among the five subtypes, the M_1_R exhibits a significant role in shaping cognitive and sensory processes [104]. Insights from M_1_R knockout (M_1_R^−/−^) mice further underscore its importance. These mice exhibit deficits in mitogen-activated protein kinase (MAPK) pathway activation, an essential signaling cascade for synaptic plasticity and cognition, particularly in hippocampal and cortical neurons [105]. Behaviorally, M_1_R^−/−^ mice perform similarly to wild-type controls in certain cognitive tasks, such as the Morris water maze, indicating that M_1_R is dispensable for basic memory formation or stability. However, under specific experimental conditions, such as non-matching-to-sample working memory tasks or fear-conditioning paradigms, these mice demonstrate significant impairments, suggesting that M_1_R is necessary for higher-order cognitive processes requiring cortical-hippocampal interactions [106].

**Figure 4:**
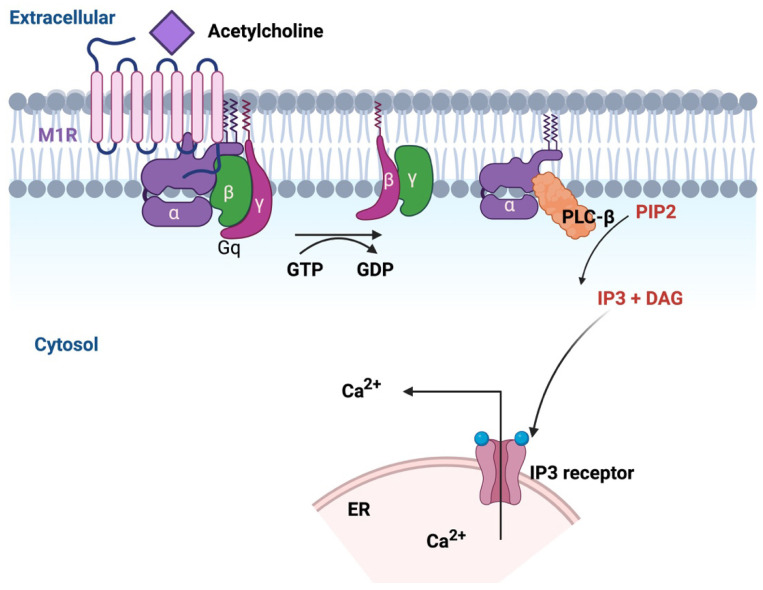
Schematic of M1 muscarinic receptor (M_1_R) signaling via the Gq protein pathway. When acetylcholine (ACh) binds to M_1_R, the Gq heterotrimeric protein (α, β, γ) becomes activated, exchanging GDP for GTP on the α-subunit. The activated Gqα then stimulates phospholipase Cβ (PLCβ), which cleaves the membrane lipid phosphatidylinositol 4,5-bisphosphate (PIP_2_) into two second messengers: inositol 1,4,5-trisphosphate (IP_3_) and diacylglycerol (DAG). IP_3_ diffuses through the cytosol and binds to IP_3_ receptors on the endoplasmic reticulum (ER), triggering Ca^2+^ ion (Ca^2+^) release into the cytosol. Increased cytosolic Ca^2+^, along with DAG, mediate downstream signaling events that lead to various cellular responses. Created with BioRender. Sanjana Chauhan. (2025) https://BioRender.com/akv9wt9 (accessed on 28 May 2025).

The M_1_R is a Gq-coupled receptor that when activated by acetylcholine (ACh) leads to breakdown of phosphatidylinositol 4,5-bisphosphate (PIP_2_) through the action of PLCβ [107] (Figure 4). This breakdown generates two important secondary messengers: inositol trisphosphate (IP_3_) and diacylglycerol (DAG). IP_3_ binds to receptors (IP_3_Rs) on the ER, stimulating the release of Ca^2+^ from the ER lumen into the cytosol [108]. Concurrently, DAG activates PKC. Together, these signaling mechanisms orchestrate a variety of neuronal processes that are required for neural development and functional maintenance, particularly axonal plasticity [109,110].

### 8.2. M_1_R Signaling for Protection Against Dying-Back Neuropathy

ACh is a neurotransmitter that modulates multiple aspects of neuronal function, including the fine-tuning of synaptic transmission and neuronal development [111]. A key role of ACh during development is regulation of axonal sprouting, during which it influences growth cone motility and cytoskeletal rearrangement [112]. Growth cones are dynamic structures that respond to extracellular signals to guide neurons toward their target destinations [113]. This motility is fundamental for the proper formation of neural circuits, as it allows for the precise wiring of neuronal connections during development. M_1_R, through its modulation of Ca^2+^ signaling within the growth cone, plays a role in controlling the dynamics of the cytoskeleton, particularly the actin filaments [113,114,115,116]. Upon ACh activation, M_1_R initiates a cascade that triggers the release of Ca^2+^, which then interacts with proteins that regulate actin polymerization. This controls the movement, extension, or retraction of the growth cone, ensuring that neurons establish appropriate synaptic connections or innervation of end-organ structures in response to environmental cues.

Collateral sprouting, a process in which axons branch and extend toward new targets in response to injury or external cues, is a cornerstone of neural plasticity and dictates fields of innervation [117]. A growing body of evidence indicates that endogenous cholinergic signaling can impose a tonic suppression on neurite outgrowth in mature neurons, a finding that has implications for therapeutic strategies targeting neuro-regenerative pathways [118]. Studies conducted across diverse models, including *Aplysia*, *Xenopus*, and mammalian embryonic neurons, indicate that ACh released from growth cones modulates Ca^2+^-dependent motility through both nicotinic and muscarinic receptors [113,116]. Activation of nicotinic receptors promotes neurite outgrowth, whereas muscarinic receptor signaling, especially as mediated by M_1_R, exerts an inhibitory effect. This suppression is largely attributed to Ca^2+^ mobilization from internal stores as well as M_1_R-mediated activation of Gα signaling, which inhibits actin filament dynamics, thereby imposing constraints on growth cone motility [115]. In mammals, sensory neurons synthesize and secrete ACh and express key components of cholinergic signaling, including the peripheral form of choline acetyltransferase (pChAT) [119]. Immunohistochemical staining of IENFs has confirmed the presence of pChAT in nerve endings [120]. These neurons also express multiple muscarinic receptors accompanied by notably low acetylcholinesterase activity [119,121]. Together, these features establish a functional endogenous cholinergic system with the potential to tonically suppress neurite outgrowth in adult sensory neurons via an autocrine and/or paracrine cholinergic mechanism. Assays using DRG-derived sensory neurons maintained in vitro replicate this mechanism, providing a robust model for studying axonal plasticity under conditions mimicking in vivo environments [122].

While muscarinic receptor signaling, particularly through M_1_R, can constrain axonal outgrowth in the peripheral nervous system, it is important to note that M_1_R activation plays distinct physiological roles in other neural contexts. In the central nervous system, M_1_R agonists enhance cognitive processes, facilitate synaptic plasticity, and promote long-term potentiation in regions such as the hippocampus and prefrontal cortex. Pharmacological activation of M_1_R has been pursued as a therapeutic approach in Alzheimer’s disease and schizophrenia, where M_1_R-selective agonists such as xanomeline and GSK1034702 have demonstrated pro-cognitive and antipsychotic effects by modulating cortical acetylcholine-dependent signaling [123]. These findings reflect the diverse, context dependent functions of M_1_R: such that while activation may be beneficial for enhancing higher-order cognitive circuits, inhibition in peripheral sensory neurons releases intrinsic growth restraints and enables structural repair following injury or disease. This dichotomy presents challenges to drug development programs and encourages use of blood–brain barrier impermeable M_1_R antagonists for use against peripheral neuropathy.

Multiple diverse muscarinic receptor antagonists have demonstrated significant promise in overcoming ACh-induced suppression of neurite outgrowth [98,115,118,124]. Of these, muscarinic toxin 7 (MT-7) and pirenzepine have been most widely studied to date. MT7, a peptide derived from green mamba snake toxin, acts as a remarkably specific negative allosteric modulator of M_1_R [125]. Pirenzepine is a competitive orthosteric antagonist that binds to the active site of M_1_R, blocking the interaction of ACh and preventing downstream signaling [126]. The potential of M_1_R antagonism to promote neurite outgrowth and mitigating neuropathic conditions has been highlighted by studies employing inhibition of M_1_R, particularly with pirenzepine and MT7 [118]. Further, M_1_R knockout (KO) mice exhibit notable resilience to diabetes-induced neuropathy, highlighting M_1_R’s pivotal role in neuroprotective pathways [118]. Intriguingly, at present there is no evidence of diabetes-induced disruption of muscarinic receptor signaling. Specifically, ChAT activity and M_1_R mRNA levels in DRG in rodent models of diabetes remain unchanged, suggesting that impaired endogenous M_1_R/acetylcholine signaling does not contribute to etiology. This raises the possibility that, although upstream M_1_R signaling remains intact, the diabetic state may compromise the intrinsic regenerative capacity of sensory neurons through mitochondrial dysfunction, metabolic stress, and impaired cytoskeletal dynamics such that even normal levels of cholinergic inhibition become disproportionately restrictive [127]. In this setting, M_1_R antagonism may relieve an otherwise tolerable inhibitory tone that becomes pathologically limiting, thereby unmasking latent growth potential, and enabling structural repair. This interpretation is consistent with broader principles of neuroplasticity in disease, where lifting inhibitory constraints can restore functional recovery under maladaptive conditions.

Building on these findings, recent studies have highlighted the importance of the AMPK/PGC-1α signaling axis in M_1_R-mediated regulation of neuronal energy homeostasis and growth [98]. Blocking M_1_R with MT7 activates the CaMKKβ-AMPK pathway in sensory neurons, which enhances mitochondrial bioenergetics. This mechanism appears spatially localized as topical application of MT7 reversed the loss of sensory nerves in the cornea in animal models of neuropathy [98,118]. In a model of CIPN when applied topically to one eye, MT7 resulted in enhanced AMPK activation and axonal repair only in the ipsilateral trigeminal ganglion, underscoring the localized effect of M_1_R antagonism on sensory fiber regeneration [98].

### 8.3. Therapeutic Implications of M_1_R Antagonism in Diverse Peripheral Neuropathy Models

The efficacy of muscarinic antagonists against indices of peripheral neuropathy has been demonstrated across multiple in vitro and in vivo model systems. In vitro studies using DRG-derived sensory neurons sourced from adult normal or diabetic rodents have shown that M_1_R antagonists enhance mitochondrial respiration and promote neurite outgrowth [98,118,128]. In these studies, total neurite outgrowth was enhanced by a range of antimuscarinic antagonists including pirenzepine, MT7 and oxybutynin. Total neurite outgrowth in this in vitro system is an indicator of collateral sprouting [122]. All sub-types of sensory neuron were responsive with the medium to larger population of neurons responding most rapidly and robustly, as previously reported for responses of sensory neurons to neurotrophic factors [129].

To date, the majority of in vivo work has been conducted in rodent models of diabetes, with rat and mouse models of STZ-induced type 1 diabetes and the db/db mouse model of type 2 diabetes used to evaluate both systemic and topical delivery of M_1_R antagonists [124,130]. The efficacy of multiple muscarinic antagonists, ranging from non-selective on-market drugs such as atropine, cyclopentolate, glycopyrrolate and oxybutynin to M_1_R selective (pirenzepine) and M_1_R specific (MT7) agents has been determined using key indices of peripheral neuropathy that reflect structural, functional, and metabolic disorders [130]. Structural endpoints include protection of small sensory nerve fiber density in skin biopsy and corneal imaging studies and axonal caliber of large, myelinated fibers [131]. Functional endpoints, such as tactile and thermal sensation provide measures of large and small fiber mediated sensory recovery, respectively, while normalization of large fiber motor and sensory nerve conduction velocity suggests a functional correlate to maintenance of axonal caliber. Metabolic endpoints, including enhanced mitochondrial respiration, ATP production, and activation of the AMPK-PGC-1α axis, highlight the improvement in bioenergetic capacity conferred by these treatments.

The therapeutic potential of M_1_R inhibition extends beyond diabetic neuropathy to other peripheral neuropathies such as those caused by chemotherapies (termed chemotherapy-induced peripheral neuropathy: CIPN) and human immunodeficiency virus (HIV) [132]. Despite differing primary etiologies, these neuropathies share similar presentations with DSPN such as distal dying-back of axons, reduced IENF density and sensory dysfunctions. Accumulating evidence suggests they may also share a pathogenic cascade that converges at impaired mitochondrial function and axonal transport deficits [41]. Mitochondrial dysfunction is a central pathogenic feature in many forms of CIPN and may arise from multiple injuries including disruption of the microtubule cytoskeleton thereby impeding axonal transport of mitochondria, direct damage to DNA, including mitochondrial DNA, that limits production of functional mitochondria and generation of a pro-oxidative and inflammatory environment that damages membranes of mature mitochondria [133]. Impaired neuronal energetic balance leading to distal axonal degeneration, sensory loss, and pain, are hallmarks of diverse peripheral neuropathies, including CIPN and DSPN. The potential for M_1_R antagonists to prevent or reverse CIPN is particularly attractive given the widespread and debilitating nature of this condition, which affects more than 80% of patients receiving standard-dose anticancer therapies [134]. A therapeutic role for M_1_R antagonists in CIPN is suggested by in vitro studies in which cultures of DRG-derived sensory neurons that were exposed to the chemotherapeutic agent oxaliplatin showed reduced neurite outgrowth that was prevented by concurrent exposure to PZ. Further, rodents with CIPN induced by paclitaxel did not develop tactile allodynia or hyperalgesia to heat when treated with systemic pirenzepine [118]. Benztropine, which inhibits both M_1_R and M_3_R, also prevented multiple indices of peripheral neuropathy in an oxaliplatin-induced model of CIPN [98,135]. A focus on the M_1_R has been emphasized by the ability of its specific antagonist, MT7, to reverse loss of corneal sensory nerves in mice with oxaliplatin-induced CIPN when the MT7 was applied topically to the eye [98]. In the context of HIV-associated neuropathy, M_1_R antagonists have demonstrated neuroprotective actions against damage induced by HIV viral proteins such as gp120 and TAT. These proteins contribute to sensory neuron toxicity by disrupting mitochondrial function and axonal transport, leading to pain, allodynia, and hypoalgesia. MT7 prevented and reversed gp120-induced reductions in neurite outgrowth in vitro and corneal nerve density loss in vivo [136]. Furthermore, in HIV-TAT transgenic mice, systemic pirenzepine administration preserved corneal innervation and protected against tactile and thermal sensory loss, likely by mitigating mitochondrial dysfunction and oxidative stress induced by TAT expression [136].

Beyond these disease-specific pathways, M_1_R antagonists exert broader neuromodulatory effects that further justify their therapeutic exploration. M_1_R signaling negatively regulates neurite extension, myelination, and synaptic plasticity [115]. Inhibiting M_1_R enhances axonal regeneration and oligodendrocyte differentiation, processes relevant across multiple neuropathies. Additionally, M_1_R antagonists modulate pain signaling and may interact with N-methyl-D-aspartate (NMDA) receptor activity, suggesting potential in broader neuropathic pain and neuroinflammation contexts.

## 9. TRPM3: A Key Player in Sensory Perception

### 9.1. Structure and Function

Transient Receptor Potential (TRP) channels are a large family of ion channels that regulate sensory perception and other physiological processes in the human body. Discovered in the late 20th century, these channels were initially identified in the fruit fly (*Drosophila melanogaster*) as components required for light perception [137]. Subsequently, numerous TRP channels have been identified in mammals, including humans, where they are involved in a broad spectrum of biological processes. These channels are present in the cell membranes of many cell types, where they act as sensors for external stimuli such as changes in temperature, chemical signals, mechanical pressure, and osmotic pressure [138]. Functionally, TRP channels are permeable to cations such as Ca^2+^ and sodium (Na^+^), allowing regulation of electrical and biochemical signals that enable cells to adapt to changes in their environment [139]. TRP channels can be classified into several subfamilies based on their structural features and functional properties. These include the TRPC (canonical), TRPV (vanilloid), TRPM (melastatin), TRPA (ankyrin), TRPP (polycystin), TRPML (mucolipin), and TRPN (*Drosophila NOMPC*, found mostly in non-mammalian species) subfamilies [140]. While all subfamilies share the common functionality of being non-selective cation channels, each has distinct characteristics. The TRPM (melastatin) group is particularly significant due to its involvement in multiple physiological and pathological processes. The TRPM family comprises eight members (TRPM1 to TRPM8), each of which has specialized functions associated with processes such as thermosensation, pain perception, insulin secretion, and ion homeostasis [141]. The general structure of TRPM channels includes six transmembrane domains with a pore-forming region between the fifth and sixth segments. Additionally, they have large intracellular domains at both the N- and C-termini, which play important roles in regulating channel activity and interacting with other cellular proteins [142]. As non-selective cation channels, TRPM members allow the passage of various ions, including Ca^2+^, Na^+^, and Mg^2+^, in response to diverse stimuli. Different TRPM channels are activated by distinct triggers. TRPM1 is crucial for retinal function and vision, while TRPM2 functions as a sensor for oxidative stress and has roles in the immune response [143]. TRPM4 and TRPM5 regulate Ca^2+^-activated cation currents, which influence electrical signaling in the heart and pancreas [144]. TRPM6 and TRPM7 are necessary for magnesium balance and are important for cellular growth and survival [143]. TRPM8 is a cold receptor, being sensitive to cool temperatures and menthol [145].

**Figure 5:**
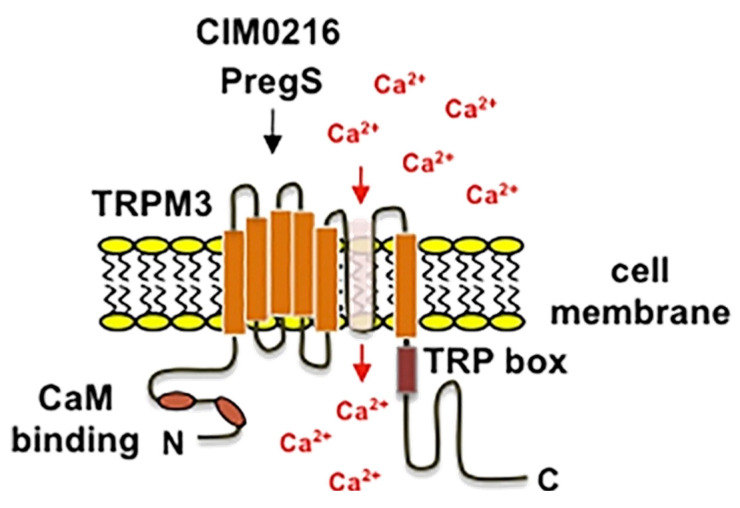
Modular structure, membrane topology, and expression of TRPM3. TRPM3 has six transmembrane domains with a pore-forming domain between transmembrane regions 5 and 6. Both the N- and C-termini project into the cytosol. The N-terminus contains two calmodulin binding sites encompassing amino acids 35–124 and 291–382. The C-terminus contains the TRP domain on the C-terminal side of the sixth transmembrane domain. Reprinted/Adapted with permission from ref. [148]. 2017 Gerald Thiel et al.

Within the TRPM family, TRPM3 has garnered attention for its distinctive functions in thermal sensation, pain detection and modulation [146,147]. TRPM3 is a non-selective cation channel that exhibits the typical structural features of TRP channels. It possesses six transmembrane domains and has a pore region that is vital for ion permeation (Figure 5).

The channel can exist in multiple isoforms due to alternative splicing, which facilitates diverse functions in different tissues. TRPM3 is highly permeable to Ca^2+^ and is broadly expressed across the body, including in the brain, sensory DRG neurons, retina, pancreatic beta cells, smooth muscle and kidney. The role of TRPM3 in thermosensation is particularly prominent [144]. In DRG neurons, TRPM3 channels are involved in nociception, particularly in responses to noxious heat and chemical stimuli [147]. TRPM3 activation also plays a significant role in inflammatory pain. For example, TRPM3-deficient mice exhibit a marked reduction in inflammatory heat hyperalgesia, indicating that TRPM3 regulates the sensitization of pain under inflammatory conditions [147]. Within the DRG, TRPM3 is expressed in a large subset of TRPV1-expressing sensory neurons, and Trpm3^−/−^ mice show reduced heat sensitivity at both cellular and behavioral levels. Interestingly, double knockout (DKO) mice lacking both TRPM3 and TRPV1 exhibit only mild heat sensitivity deficits, suggesting compensatory mechanisms involving other heat-sensitive channels [149]. However, triple knockout mice lacking TRPM3, TRPV1, and TRPA1 display profound deficits in heat sensing, confirming the complementary roles of these channels in acute noxious heat detection and their interplay in maintaining thermosensation. When exposed to inflammatory stimuli, TRPM3 channels become sensitized, leading to enhanced pain perception. This makes TRPM3 a potential therapeutic target for treating inflammatory pain disorders. For instance, antagonists that inhibit TRPM3 activation alleviate thermal and mechanical hyperalgesia, suggesting that TRPM3 inhibitors could provide relief in conditions such as neuropathic and inflammatory pain [150].

Beyond its role in nociception, TRPM3 is also involved in metabolic processes, particularly in pancreatic beta cells [151]. In these cells, TRPM3 activation by pregnenolone sulfate (PS) enhances glucose-induced insulin secretion, linking this ion channel to glucose metabolism and insulin regulation. This suggests that TRPM3 could play a role in metabolic disorders such as diabetes, where impaired insulin secretion occurs. Emerging evidence also connects TRPM3 dysfunction to neurodevelopmental disorders, particularly developmental and epileptic encephalopathies (DEE) [152], which are characterized by epilepsy, intellectual disabilities, and musculoskeletal abnormalities [153,154]. Rare de novo mutations in the TRPM3 gene have been identified in individuals with DEE, revealing gain-of-function effects that result in increased basal activity of the channel. Functional studies in mammalian cells and frog oocytes demonstrate that these mutations induce greater TRPM3 sensitivity to heat and activators, such as PS, while also elevating baseline Ca^2+^ permeability; even in the absence of stimuli. This aberrant Ca^2+^ influx leads to intracellular Ca^2+^ overload. Patients with DEE-associated TRPM3 mutations exhibit a spectrum of neurological and developmental symptoms, including global developmental delay, epilepsy, altered pain perception, and cerebellar abnormalities such as ataxia and hypotonia. Notably, the antiseizure medication primidone, a TRPM3 antagonist, reduced the basal hyperactivity of mutant channels, providing a potential therapeutic approach for managing DEE-related symptoms [154].

### 9.2. TRPM3 Pharmacology

The pharmacological modulation of the TRPM3 channel offers a compelling avenue to explore its multifaceted roles in sensory, metabolic, and neurological systems. TRPM3 channels respond to diverse endogenous and synthetic modulators that influence its gating mechanisms, specificity, and physiological outcomes [155]. These modulators, encompassing both agonists and antagonists, provide tools for understanding the channel’s intricate pharmacology and provide potential therapeutic applications. TRPM3 activation involves conformational changes in channel structure, particularly at the voltage-sensing domain (VSD) formed by transmembrane segments S1–S4 and the pore domain composed of S5 and S6 [156]. These structural shifts lead to opening of the ion-conducting pore, allowing influx of cations such as Ca^2+^, Na^+^ and Mg^2+^. PS, an endogenous neurosteroid, and nifedipine, a synthetic ligand, are among the classical activators of TRPM3 [147,155,157,158]. These agents predominantly induce Ca^2+^ influx through the central pore, characterized by an outwardly rectifying current-voltage (I–V) relationship. However, TRPM3’s unique ability to support two distinct ion permeation pathways sets it apart from other TRP channels. There exists a secondary, non-canonical ion permeation pathway in TRPM3, that becomes active under specific conditions [159]. Combined stimulation with PS and clotrimazole (Clt), an antifungal agent, leads to the simultaneous activation of the canonical Ca^2+^-permeable pore and the alternative pathway. The latter mediates an inwardly rectifying current predominantly carried by monovalent cations such as Na^+^. This non-canonical pathway exhibits unique properties, including resistance to Ca^2+^-dependent desensitization and insensitivity to conventional pore blockers such as La^3+^ [155,159].

CIM0216, a synthetic agonist with exceptional potency, provides a valuable tool for dissecting TRPM3’s dual pore functionality [155]. Unlike PS, which requires concurrent exposure to Clt for full activation of the non-canonical pathway, CIM0216 independently activates both permeation pathways, inducing robust inward and outward rectifying currents. Interestingly, CIM0216 lacks the acidic substituents critical for PS-mediated activation, suggesting a distinct binding mechanism. Its structure, consisting of three aromatic rings around a central linking moiety, shares basic similarities with Clt, potentially enabling its interaction with specific domains of TRPM3 that regulate the non-canonical pore. The dual activation by CIM0216 significantly augments intracellular Ca^2+^ signals, particularly in excitable cells such as sensory neurons and pancreatic beta cells. This mechanism facilitates Na^+^-driven membrane depolarization, which in turn activates voltage-gated Ca^2+^ channels, amplifying Ca^2+^ influx and enhancing downstream signaling, including vesicular release.

TRPM3 antagonists such as isosakuranetin and primidone counteract channel activation by blocking ion influx or altering channel conformation [150,160]. Isosakuranetin, a flavonoid with high specificity for TRPM3, effectively blocks both the canonical and non-canonical pathways by inhibiting ion flux induced by endogenous and synthetic agonists [160]. Its specificity for TRPM3 over other TRP channels makes isosakuranetin a valuable tool for studying the role of TRPM3 in sensory and inflammatory processes. Similarly, the anticonvulsant drug primidone has shown efficacy in inhibiting TRPM3-mediated Ca^2+^ influx, reducing neurogenic inflammation and pain [150].

### 9.3. GPCR Regulation of TRPM3 Channels

One of the unique features of TRPM3 is modulation by GPCRs through intricate mechanisms that influence function in pain, inflammation, and other sensory processes. The interaction between TRPM3 and GPCR pathways is one mechanism through which opioid analgesics exert their peripheral effects. Activation of the opioid μ receptor, a Gi-coupled GPCR, drives the release and binding of Gβγ to TRPM3 effectively gating the channel and preventing its activation. This offers a molecular basis for how opioids can modulate pain by direct interaction with primary sensory neurons [161,162]. Similarly, Gq- and Gs-coupled receptors, including prostaglandin EP2 and bradykinin B2 receptors, inhibit TRPM3, and highlight the channel’s central role in nociceptive signaling. TRPM3 regulation extends beyond Gβγ-mediated inhibition. The channel is dynamically modulated by phosphoinositides, particularly PIP_2_ [163,164]. PIP_2_ not only enhances TRPM3 activity but also plays a pivotal role in promoting recovery from channel desensitization. This recovery is facilitated by phosphatidylinositol kinases (PI-Ks), which resynthesize PIPs in the plasma membrane, thus reactivating TRPM3 functionality. Among the phosphoinositides, phosphatidylinositol (3,4,5)-trisphosphate (PIP_3_) and PIP_2_ exhibit the highest efficacy in potentiating TRPM3 activity, while phosphatases that deplete these lipids act as inhibitory factors. This lipid-mediated regulation underscores the importance of maintaining appropriate PIP levels to ensure TRPM3 functionality.

**Figure 6:**
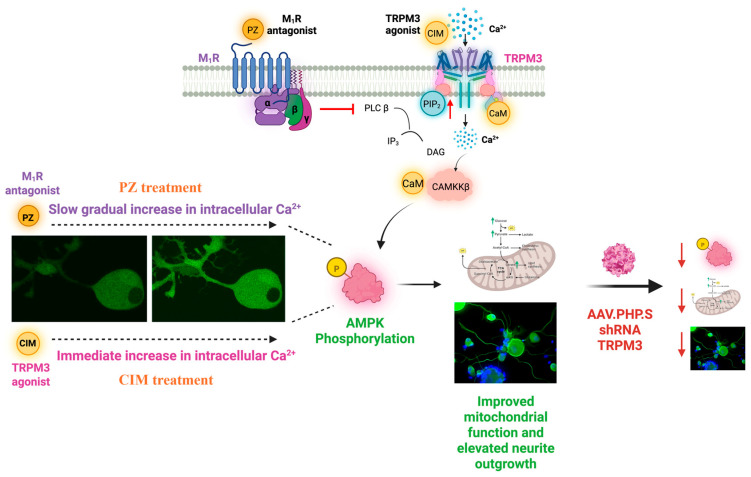
Schematic representation of TRPM3 activation and its role in M_1_R antagonist-mediated neuroprotection. M_1_R antagonism (PZ) prevents PIP_2_ hydrolysis, leading to a gradual increase in intracellular Ca^2+^, whereas TRPM3 agonists (CIM0216/PS) induce an immediate Ca^2+^ influx. Increased Ca²⁺ activates CaMKKβ-mediated AMPK phosphorylation, enhancing mitochondrial function and neurite outgrowth. Dashed lines indicate connections between representative cellular images and their respective Ca^2+^ signaling outcomes. Right panel shows that TRPM3 knockdown reduces AMPK activation and mitochondrial bioenergetics and metabolism. Created with BioRender.Sanjana Chauhan. (2025) https://BioRender.com/jkmai3w (accessed on 28 May 2025).

Recent findings from our laboratory have identified a unique aspect of this regulation, whereby M_1_R antagonism elevates PIP_2_ levels and activates TRPM3 channels [165]. This study established a mechanistic link between M_1_R antagonism, increased PIP_2_ availability, and subsequent TRPM3 activation. Specifically, we demonstrated that M_1_R inhibition leads to measurable elevation of PIP_2_, and this phospholipid accumulation correlates with enhanced TRPM3 channel activity. These results align with existing literature showing that elevated PIP_2_ potentiates TRPM3 by stabilizing its open conformation and promoting recovery from desensitization [164]. While our data provided experimental evidence for this signaling axis in a defined model system, we acknowledge that further validation is needed in broader physiological and pathological contexts. Importantly, pharmacological inhibition or in vitro knockdown of TRPM3 abolished the neuroprotective effects of M_1_R antagonism, confirming that functional TRPM3 channels are essential mediators of this response (Figure 6) [165]. These findings support the existence of a functional M_1_R–PIP_2_–TRPM3 signaling pathway with potential therapeutic relevance.

### 9.4. Effect of TRPM3 Activation

Activation of TRPM3 by physiological stimuli or pharmacological agents initiates a robust influx of Ca^2+^, surpassing that observed through depolarization-induced pathways such as voltage-gated Ca^2+^ channels [155]. Ca^2+^ entry through TRPM3 channels triggers the binding of Ca^2+^ to calmodulin, a key Ca^2+^ sensor that modulates TRPM3 activity [166]. Calmodulin directly interacts with TRPM3 channels, facilitating their activation and subsequent intracellular signaling [167]. Through its EF-hand motifs, calmodulin binds Ca^2+^ ions with the Ca^2+^-bound form modulating TRPM3 activity and downstream signaling pathways. Importantly, this interaction serves as a feedback mechanism: Ca^2+^ influx through TRPM3 activates calmodulin, which in turn activates calcineurin, a Ca^2+^/calmodulin-dependent phosphatase that acts as a negative regulator of TRPM3 signaling [166].

Following TRPM3 activation, mitochondria rapidly buffer approximately 40% of the incoming Ca^2+^ [168]. This buffering leads to a steady-state elevation of mitochondrial Ca^2+^ levels, a phenomenon that is further amplified by nerve growth factor (NGF), which enhances TRPM3 activity and slows the clearance of intracellular Ca^2+^. The delayed recovery of cytosolic Ca^2+^ extends the duration of Ca^2+^-dependent signaling cascades, underscoring the unique role of TRPM3 in modulating intracellular Ca^2+^ homeostasis. This sustained Ca^2+^ signaling and buffering of Ca^2+^ within the mitochondrial matrix has the potential to activate enzymes of the TCA cycle, including pyruvate dehydrogenase (PDH) and isocitrate dehydrogenase (IDH), which in turn accelerate ATP production to meet the high metabolic demands of neurons.

Recent findings from our laboratory using sensory neurons from adult rodents [165] have confirmed reports [147,159] that pharmacological activation of TRPM3 using specific agonists, such as CIM0216 and PS, stimulates increased intracellular Ca^2+^. Our study also provides the first evidence that activation of TRPM3 by its specific agonists CIM0216 and PS leads to activation of the CaMKKβ pathway and time dependent phosphorylation of AMPK in DRG-derived sensory neurons (Figure 6) [165]. While prior research in clear cell renal carcinoma cells identified a connection between TRPM3 activation, CaMKKβ, and AMPK signaling [169], its relevance to neuronal function, metabolism, and mitochondrial regulation had not been previously established. As described above, neurons rely on finely tuned Ca^2+^ signaling to regulate mitochondrial activity and optimize energy production [170]. In sensory neurons, mitochondria are strategically positioned near the plasma membrane, allowing rapid Ca^2+^ buffering when Ca^2+^ enters through ion channels, such as TRPM3 [168].

Although sustained elevations in cytosolic Ca^2+^ are deleterious in DSPN due to impaired mitochondrial buffering and oxidative stress, TRPM3-mediated Ca^2+^ influx occurs under controlled conditions that are transient, spatially confined, and rapidly buffered by perimembranous mitochondria. We propose this localized signal is sufficient to activate CaMKKβ without triggering global Ca^2+^ toxicity. Notably, CaMKKβ activation by intracellular Ca^2+^ exerts neuroprotective effects in other injury models, such as stroke, where it enhances transcriptional activation, preserves blood–brain barrier integrity, and suppresses neuroinflammation, ultimately supporting neuronal survival [171]. This mechanistic distinction reconciles the earlier described pathological Ca^2+^ dyshomeostasis with the therapeutic rationale for targeting TRPM3 to activate regenerative signaling in DRG neurons.

TRPM3 dependent Ca^2+^ signaling activates the CaMKKβ/AMPK pathway, enhancing mitochondrial function and promoting neurite outgrowth. TRPM3 knockdown using AAV-PHP.S shRNA reduces AMPK phosphorylation and metabolic activity, impairing neuronal growth and repair, indicating that the M_1_R-mediated effects are TRPM3-dependent. Quantitative imaging assays and mass spectrometry-based metabolic profiling have revealed that TRPM3 activation enhanced glycolysis and TCA cycle metabolite levels in DRG neurons [165]. This metabolic shift suggested that TRPM3-mediated Ca^2+^ influx not only improved mitochondrial function but also stimulated glucose metabolism to meet the sustained energy demands of neurons. It is proposed that the mechanism of AMPK-dependent mitochondrial enhancement and augmentation of neuronal metabolism involved activation of the co-transcriptional activator PGC-1α and the subsequent stimulation of gene expression of components of the respiratory chain and antioxidant pathways [88]. Further supporting this notion, activation of TRPM3 enhanced neurite outgrowth in adult DRG-derived sensory neurons [165]. These observations directly link TRPM3 activation to neuroprotective mechanisms.

In addition to its pro-regenerative effects, TRPM3 is a known sensor of noxious heat and an important contributor to inflammatory pain. In vivo studies have shown that TRPM3 is upregulated in models of CIPN [146], and its genetic ablation or pharmacological blockade reduces mechanical hypersensitivity. Furthermore, TRPM3 activation enhances presynaptic transmission in nociceptive circuits of the spinal dorsal horn, and inflammatory mediators can sensitize TRPM3 via post-translational modifications [172]. These findings underscore the risk that TRPM3 agonists may exacerbate pain in patients with neuropathic conditions. However, although our recent study did not evaluate nociceptive thresholds or pain behaviors, our mechanistic data [165] demonstrate that TRPM3-mediated Ca^2+^ signals are spatially restricted and rapidly buffered, limiting the risk of broad nociceptor activation. Localized (e.g., topical or intradermal) delivery and sub-nociceptive dosing of TRPM3 agonists may enable targeted activation of pro-regenerative pathways while avoiding widespread sensory sensitization. Future studies should focus on defining this window and systematically testing the safety and efficacy of TRPM3-targeted therapies in relevant models of neuropathic pain.

## 10. The Therapeutic Potential of TRPM3 and M_1_R-Targeted Therapies

Although the clinical presentation of peripheral neuropathy can vary widely among patients with different underlying conditions, mitochondrial dysfunction has emerged as a central contributing factor across many neuropathies [41,61]. This dysfunction contributes to the retraction or degeneration of peripheral sensory terminals from their target tissues, ultimately resulting in sensory deficits. Evidence from human skin biopsies shows a marked reduction in mitochondrial density within IENF of patients with early signs of neuropathy [63]. Targeting mitochondrial dysfunction through modulation of TRPM3 and M_1_R signaling holds promise as a therapeutic strategy as it offers an approach that goes beyond symptom management to actively drive nerve repair and regeneration [165]. This mechanism-driven intervention strategy represents a shift in therapeutic design from palliative symptom relief to true disease modification by restoring bioenergetic balance, halting axonal degeneration, and promoting functional reinnervation. Our in vivo studies have demonstrated that treatment with the M_1_R antagonist pirenzepine in rodent models of type 1 and type 2 diabetes resulted in nerve protection and repair [98,118]. These effects were closely associated with restoration of AMPK activity and improvement of mitochondrial dysfunction in DRG-derived sensory neurons. Pirenzepine treatment in rodent models of type 1 and type 2 diabetes not only prevented further nerve degeneration but also reversed key indices of neuropathy, including the loss of IENF and sensory function.

The translational potential of M_1_R antagonists as a treatment for diabetic neuropathy has recently been assessed in two clinical studies. The first involved topical delivery of oxybutynin, a non-selective M_1_R antagonist used clinically to treat overactive bladder. Preclinical studies confirmed that, in diabetic rodents, oxybutynin replicated the efficacy against IENF loss previously reported for other M_1_R antagonists [128]. In a subsequent randomized, double-blind, placebo-controlled trial in patients with type 2 diabetes and confirmed peripheral neuropathy, subjects treated with 3% oxybutynin applied daily for 5 months to the upper arms, stomach or calves and upper feet in a rotating fashion demonstrated a significant increase in IENF density compared to pre-treatment values, whereas those treated with placebo did not [128]. This replicates preclinical findings and suggests change in IENF density as an objective biometric parameter for demonstrating regenerative growth of small sensory fibers. Increased IENF density was accompanied by improvement in other more subjective measures of neuropathy that may or may not be directly related to nerve regeneration or protection, including reduced neuropathic pain and improved quality-of-life scores [128]. These improved endpoints included NIS total score, NTSS-6 pain quality, NRS pain feet and legs, and Norfolk quality of life-diabetic neuropathy (QOL-DN) including total score and physical/large fiber function. However, not all indices of neuropathy measured showed significant improvement following oxybutynin treatment. Most notably, large fiber nerve conduction velocity (NCV), which has been used as a primary indicator of therapeutic efficacy in large scale clinical trials against diabetic neuropathy, was not impacted by treatment. The discord between the response of IENF density and NCV to oxybutynin may illustrate the need for a more refined approach in selecting primary efficacy end points in registration trials, as direct measures of large fiber function such as NCV need not reflect efficacy of agents that selectively impact small sensory fibers. Alternately, the discord may simply reflect assay sensitivity or dose, efficacy variability for different fiber types, echoing our early preclinical studies in which low systemic doses of M_1_R antagonists were effective on small fiber structure and function but not large fiber function, whereas, higher doses were effective on both fiber types [124].

Most recently, a randomized Phase 2a, double-blind, placebo-controlled, clinical trial was conducted across 5 university centers in Canada [131]. A topical formulation of pirenzepine (2% and 4% doses; n = 58) was self-administered daily to the lower limb by persons with type 2 diabetes and mild-moderate neuropathy for a 24-week period. Multiple biometric (IENF density, large fiber nerve conduction velocity, sensory perception) and patient reported outcomes were monitored (including the Toronto Clinical Neuropathy Score (TCNS) symptom score and Norfolk QOL-DN). Of these, change in IENF density, representing small fiber regenerative growth from the dermis, was designated as the primary end point as it most directly reflects the presumed mechanism of action of pirenzepine, representing small fiber regenerative growth from the dermis into the epidermis. The least squares mean difference in change from baseline to week 24 in the IENF density, the primary endpoint, at the ankle was 2.32 (*p* = 0.006) in the pirenzepine 4% group; 1.50 (*p* = 0.048) in the pirenzepine 2% group and −0.71 (*p* = 0.39) in placebo patients. The change in IENF density at the ankle was statistically significant in the combined pirenzepine groups compared to placebo (*p* = 0.012). As with the clinical study using oxybutynin, change in IENF density once again served as a reliable biomarker of sensory nerve regenerative growth. In contrast to the oxybutynin study described above, pirenzepine also showed a non-significant trend for increase in sural nerve large fiber NCV when both dose groups were combined (1.89 m/s vs. placebo). This may reflect an improved local dosing regime of a more selective M_1_R antagonist. It is notable that preclinical studies in diabetic rats treated with the same topical formulation of pirenzepine, as used in the clinical trial, demonstrated efficacy against reduced caliber of large, myelinated axons [128]. As axonal caliber is a major determinant of NCV and reduced caliber contributes to NCV slowing, the improved NCV of diabetic patients treated with pirenzepine may represent a functional manifestation of structural neuroprotection by this anti-muscarinic agent.

Of the non-biometric endpoints assessed, there was a 10.4- point improvement in the Norfolk QOL-DN score in the combined treatment groups over placebo (*p* < 0.001) in the per-protocol analysis set. Other subjective outcomes were less responsive; for example, the quantitative cooling detection thresholds, quantitative vibration perception thresholds and the visual analog scale (VAS) were unaffected. Despite the limited efficacy on subjective patient reported outcomes, which it should be noted were collected under challenging conditions for subjects and clinical staff during the first years of the 2020 COVID-19 epidemic, this is the first study in humans with diabetic neuropathy where selectively targeting a specific receptor has objectively promoted regeneration of distal sensory axons to improve a major pathological feature of early diabetic neuropathy.

M_1_R antagonism potentially offers broad therapeutic applicability to diverse peripheral neuropathies, including diabetic neuropathy, CIPN, and HIV-associated neuropathy. The clinical potential of M_1_R antagonism is currently under investigation in ongoing trials of pirenzepine in multiple patient populations. In a 16-week study (NCT05005078), pirenzepine is being tested in patients with HIV-associated distal sensory polyneuropathy while another clinical study (NCT05488873) is evaluating efficacy in oncology patients with CIPN. The well-characterized safety profile of pirenzepine, supported by decades of clinical use, positions pirenzepine as a promising therapy that could translate rapidly into clinical practice

As outlined above, TRPM3 represents a compelling target for treating neuropathic disease through its role in modulating mitochondrial Ca^2+^ dynamics and bioenergetics. By facilitating robust mitochondrial Ca^2+^ loading [168], TRPM3 activation enhances ATP production and sustains mitochondrial function under stress conditions. Given its ability to regulate processes such as mitochondrial respiration and nerve repair [165], TRPM3 also plays a role in pathways linked to pain sensitization, offering the dual benefit of addressing sensory deficits and modulating pain [146]. With M_1_R antagonism demonstrating effectiveness across multiple neuropathies, TRPM3, as a downstream target of M_1_R activity, represents a promising alternative and/or complementary target for nerve repair and functional recovery in various neuropathic conditions. Activation of TRPM3 enhances oxidative phosphorylation and ATP production, while simultaneously activating the CaMKKβ–AMPK signaling axis [165]. AMPK activation promotes mitochondrial biogenesis, cytoskeletal remodeling, and axonal elongation—processes essential for the restoration of functional innervation [173]. M_1_R antagonism complements this regenerative response by relieving tonic cholinergic inhibition on neurite outgrowth and stabilizing neuronal excitability via Kv7-mediated M-current enhancement [118,174]. Together, these integrated mechanisms support structural repair of damaged nerve fibers, distinguishing this approach from conventional symptomatic treatments. By addressing core deficits in bioenergetics, axonal degeneration, and synaptic connectivity, M_1_R/TRPM3 modulation represents a significant paradigm shift in the treatment of peripheral neuropathy—one that redefines therapeutic success in terms of regeneration and long-term functional recovery. Unfortunately, while the primary TRPM3-related compounds used in preclinical studies, such as CIM0216 and PS, have provided important mechanistic insights, poor pharmacokinetic properties limit their translational potential. For example, CIM0216, despite being a small molecule with a molecular weight comparable to pirenzepine, is highly lipophilic and poorly soluble, making it unsuitable for aqueous formulations or topical slow-release delivery systems. The emerging therapeutic potential of targeting TRPM3 may provoke generation of new, clinically viable agents.

In addition to the TRPM3-mediated mechanism described above, M_1_R antagonists have been shown in other systems to engage multiple TRPM3-independent pathways that may contribute to neuroprotection and repair. These include enhancement of the Kv7.2/7.3-mediated M-current [174], leading to neuronal hyperpolarization and suppression of spontaneous excitability; β-arrestin-biased activation of ERK1/2 via casein kinase 2 that promotes neurite outgrowth independent of G protein signaling (unpublished; paper accepted subject to further review); promotion of oligodendrocyte precursor cell differentiation and remyelination in CNS demyelinating models [175] and attenuation of nitric oxide production and lipid peroxidation in oxidative injury paradigms [176]. Activation of one or more of these mechanisms may evoke a multifaceted pharmacological profile for M_1_R antagonists that positions them as regenerative agents across diverse neuropathological contexts.

It is important to consider potential on-target risks associated with systemic modulation of these pathways. Both M_1_R and TRPM3 are expressed in other tissues, including the central nervous system, pancreas, and kidney. M_1_R antagonism may pose risks of cognitive or anticholinergic side effects [177] unless limited by use of molecules such as pirenzepine that do not enter the CNS, while TRPM3 activation has been implicated in pancreatic beta-cell Ca^2+^ handling, insulin secretion, and thermal nociception [178,179]. Although localized delivery can minimize such risks, broader clinical application will require the development of peripherally selective compounds or delivery strategies that limit off-target engagement. Addressing these translational challenges will be critical for the safe and effective deployment of M_1_R and TRPM3 modulators as therapies for peripheral neuropathy.

## 11. Future Directions

As discussed above, existing M_1_R antagonists are currently under clinical investigation for repurposing as therapy for peripheral neuropathy, although there is considerable room for advancing novel second generation agents that offer more precise targeting. To translate TRPM3-based therapies into the clinic, future drug development must prioritize the identification or design of novel TRPM3 agonists with enhanced solubility, bioavailability, and stability. Given the broad tissue distribution of TRPM3, these compounds would need to be optimized for safe and sustained delivery, potentially via topical or localized routes that minimize systemic exposure and off-target effects. There is also a rationale for developing peptide-based or protein modulators with higher selectivity for peripheral sensory neurons, as well as exploring nanocarrier-based formulations to enable controlled release. While TRPM3-targeting agents have not yet reached clinical evaluation, the dependence of M_1_R antagonist-mediated nerve repair on TRPM3 activation provides strong justification for further development. The clinical progress and safety profile of M_1_R antagonists offer a useful precedent, supporting the feasibility of TRPM3 modulation as a complementary or standalone strategy for promoting nerve regeneration and functional recovery in neuropathic conditions.

## Abbreviations

The following abbreviations are used in this manuscript:

ACh	Acetylcholine
AGE	Advanced glycation end-product
AMPK	AMP-activated protein kinase
ATP	Adenosine triphosphate
CaMKKβ	Ca^2+^/calmodulin-dependent protein kinase kinase β
CBS	Cystathione β-synthase
CCM	Corneal confocal microscopy
CIPN	Chemotherapy-induced peripheral neuropathy
Clt	Clotrimazole
COX-2	Cyclooxygenase-2
DAG	Diacylglycerol
DEE	Developmental and epileptic encephalopathies
DM	Diabetes mellitus
DRG	Dorsal root ganglion
DSPN	Diabetic sensorimotor polyneuropathy
ER	Endoplasmic reticulum
ETC	Electron transport chain
GLUT3	Glucose transporter type 3
GPCR	G protein-coupled receptor
HDL	High-density lipoprotein
HFD	High-fat diet
HIV-DSP	HIV-associated distal sensory polyneuropathy
HSP	Heat shock proteins
IDF	International Diabetes Federation
IDH	Isocitrate dehydrogenase
IENF	Intraepidermal nerve fiber
IL-6	Interleukin-6
IP3	Inositol trisphosphate
IP3R	Inositol 1,4,5-trisphosphate receptor
LDL	Low-density lipoprotein
LKB1	Liver kinase B1
M1R	Muscarinic acetylcholine type 1 receptor
MAM	Mitochondria-associated membrane
MAPK	Mitogen-activated protein kinase
MCU	Mitochondrial Ca^2+^ uniporter
MDA	Malondialdehyde
MT7	Muscarinic toxin 7
mtTFA	Mitochondrial transcription factor A
NADPH	Nicotinamide adenine dinucleotide
NF-κB	Nuclear factor κ-light-chain-enhancer of activated B cells
NMDA	N-methyl-D-aspartate
NRF	Nuclear respiratory factor
pChAT	Peripheral form of choline acetyltransferase
PDH	Pyruvate dehydrogenase
PGC-1α	Peroxisome proliferator-activated receptor-γ coactivator-1α
PIP2	Phosphatidylinositol 4,5-bisphosphate
PIP3	Phosphatidylinositol (3,4,5)-trisphosphate
PKC	Protein kinase C
PLC	Phospholipase C
PS	Pregnenolone sulfate
ROS	Reactive oxygen species
RyR	Ryanodine receptor
SERCA	Sarco/endoplasmic reticulum Ca^2+^ ATPase
STZ	Streptozotocin
TCA	Tricarboxylic acid
TNF-α	Tumor necrosis factor-α
TNF-β	Tumor necrosis factor-β
TRP	Transient receptor potential
TRPM3	Transient receptor potential melastatin-3
UCP	Uncoupling protein
VDAC	Voltage-dependent anion channel
VSD	Voltage-sensing domain
β-SID	β-subunit interacting domain
